# The real life experience goes on: update after 4 years on the first cohort treated with lanadelumab at our center

**DOI:** 10.3389/fimmu.2024.1405317

**Published:** 2024-05-10

**Authors:** Thomas Buttgereit, Carolina Vera Ayala, Seda Aykanat, Karsten Weller, Annika Gutsche, Marcus Maurer, Markus Magerl

**Affiliations:** ^1^ Institute of Allergology, Charité – Universitätsmedizin Berlin, Corporate Member of Freie Universität Berlin and Humboldt-Universität zu Berlin, Berlin, Germany; ^2^ Fraunhofer Institute for Translational Medicine and Pharmacology ITMP, Immunology and Allergology, Berlin, Germany

**Keywords:** lanadelumab, hereditary angioedema, real-life, control, quality of life

## Abstract

**Introduction:**

Lanadelumab is a first-line long-term prophylaxis (LTP) in hereditary angioedema (HAE). Real-life data on its long-term efficacy and safety are limited. It is unknown whether patients using lanadelumab need short-term prophylaxis (STP).

**Objectives:**

To provide 4-year follow-up data for our first 34 patients treating with lanadelumab.

**Methods:**

Patients were assessed for their current injection interval, attacks, treatment satisfaction, disease control (AECT), quality of life impairment (AE-QoL), events that can induce attacks, and the use of STP since the start of their treatment with lanadelumab.

**Results:**

Of 34 patients who started lanadelumab treatment, 32 were still using it after 4 years, with a median injection interval of 33 (range 14-90) days. HAE patients (n=28) reported longer intervals, i.e. 35 (14-90) days, than patients with angioedema due to acquired C1 inhibitor deficiency (n=4, 23 (14-31) days). With their current injection intervals, used for a mean duration of 29 ± 17 months, patients reported a yearly attack rate of 0.3 ± 0.1. More than 70% of patients were attack-free since starting their current injection interval. All patients reported well-controlled disease, i.e. ≥10 points in the AECT; 21 patients had complete control (16 points). AE-QoL scores improved further compared to our initial report, most prominently in the fears/shame domain (-6 points). Treatment satisfaction was very high. No angioedema occurred after 146 of 147 potentially attack-inducing medical procedures without STP.

**Conclusions:**

Our results demonstrate the long-term efficacy and safety of lanadelumab in real-life and question the need for STP in patients who use effective LTP.

## Introduction

C1 inhibitor (C1INH) deficiency leads to insufficient inhibition of the kinin system. This allows kallikrein to excessively release bradykinin, which subsequently increases vascular permeability and leads to the development of angioedema (1). The most common reason for C1INH deficiency is hereditary angioedema (HAE-C1INH), a rare genetic disease caused by a mutation in the SERPING1 gene (2). Much more rarely, recurrent angioedema due to C1INH deficiency is acquired, i.e., AAE-C1INH, often in the context of an underlying lymphoproliferative disease or the corresponding immune reaction against it (3). Angioedema attacks in patients with C1INH deficiency are unpredictable. Depending on their localization, swellings can be function-restricting, painful, disfiguring or even life-threatening, having a high impact in patients’ quality of life (QoL).

For the treatment of C1INH deficiency, on-demand treatment (ODT), short-term prophylaxis (STP) and long-term prophylaxis (LTP) treatment options are available. The international guideline for HAE recommends icatibant, plasma-derived (pd) or recombinant C1INH and ecallantide as first-line options for ODT of HAE attacks (2). For STP, pdC1INH is recommended before medical, surgical or dental procedures as well as exposure to other events that come with the risk of inducing an attack. In recent years, more and more HAE patients have turned to the use of modern LTP, to prevent the occurrence of swellings. The international guideline supports this approach by calling for the goals of treatment to be “complete disease control” and the “normalization of life”, which can currently only be achieved by the use of LTP (2, 4). In February 2019, lanadelumab, a monoclonal antibody against plasma kallikrein, became available in Germany for LTP in patients with HAE from the age of 12 in a dosage of 300 mg as a subcutaneous injection every 2 weeks, but longer treatment intervals can be considered if there are no symptoms (5). Lanadelumab has shown unprecedented efficacy with a favorable safety profile in long-term clinical studies (6, 7). Very recently, lanadelumab has been approved for LTP in patients from 2 years of age (8).

In 2021, we reported on the first 34 patients with recurrent angioedema due to C1INH deficiency, 30 patients with HAE-C1INH and 4 patients with AAE-C1INH, who were treated with lanadelumab at our Angioedema Center of Reference and Excellence (ACARE) (9, 10). Specifically, we described the safety profile of lanadelumab and its marked effects on disease control and disease-related quality of life as well as the individualization of lanadelumab therapy by gradually increasing the duration of injection intervals. At the time, patients were using lanadelumab, on average, at intervals of 30 days, with many patients still in the process of increasing the duration of their treatment intervals. In the meantime, treatment with lanadelumab has become routine clinical practice, and the drug has been introduced in numerous other patients using the described protocol for interval prolongation.

As of now, real-life data on the long-term efficacy and safety of lanadelumab are limited. To address this gap of knowledge, we assessed the first 34 patients who started lanadelumab treatment at our ACARE for their current injection interval, attacks, treatment satisfaction, disease control (AECT), quality of life impairment (AE-QoL), events that can induce attacks, and the use of STP since the start of their treatment with lanadelumab four years ago.

## Methods

### Patients

Of the 34 patients (median age: 47 years, range 19–78 years) who initiated LTP with lanadelumab at our ACARE from February 2019 to April 2020, 30 had HAE-C1INH and four had AAE-C1INH. Before switching to lanadelumab, 10 patients used LTP with pdC1INH intravenously (on average 5 breakthrough attacks per month), 11 patients administered pdC1INH prophylaxis subcutaneously (on average 4 breakthrough attacks per month), and 1 patient received LTP with tranexamic acid orally (6 breakthrough attacks per month). Six patients had used only ODT (with icatibant, on average 6 attacks per month). A further 6 patients were roll-overs (RO) from the open-label extension (OLE). All patients started lanadelumab treatment using the Berlin protocol, as previously described (9). In the further course, all patients were seen at the ACARE Berlin, in person at regular intervals of around 6 to 12 months, depending on their disease burden, personal situation and individual need for consultation. During the consultations, all patients were assessed for their last attack, the number of attacks since the start of treatment with lanadelumab, the current injection interval, patient reported outcomes (PROMs), the use of STP, medical procedures that can induce attacks, and satisfaction with the treatment.

Two of the 34 patients discontinued treatment with lanadelumab and returned to ODT at their request. The first patient, a 39-year old female, discontinued treatment with lanadelumab every 35 days at the end of 2021 with the explanation that she faced fewer attack triggers due to a major career change. At the last contact in June 2023, she reported that she only had two attacks in the last year. The other patient with HAE, a 37-year old female, discontinued monthly injections of lanadelumab in November 2022 due to self-reported intolerance reactions to various foods, which manifested as the occurrence of angioedema and wheals. Under the additional diagnosis of chronic spontaneous urticaria, treatment with omalizumab was initiated in June 2023, and the patient is still symptom-free until today. These two patients were not included in the following analysis. The present analysis includes 32 patients, 28 patients with hereditary angioedema due to C1 inhibitor deficiency (21 female, median age 48 years, range 22-82), and four patients (all female) with acquired C1 inhibitor deficiency (median age 66 years, range 63-72). Of the 32 patients, 14 live in Berlin or in the Berlin public transport area, the remaining patients live a median of 184 km from our ACARE (range 49-371).

### Patient reported outcome measures

Between August and November 2023, patients completed the Angioedema Control Test (AECT) and the Angioedema Quality of Life questionnaire (AE-QoL).

The AECT is a validated, retrospective 4-item (each scored 0-4 points) questionnaire that assesses patients’ disease control over recurrent angioedema symptoms in the previous 4 or 12 weeks. The cutoff value of <10 points separates those with insufficiently controlled disease (minimum 0 points) from those with well-controlled disease (maximum 16 points) (11).

The AE-QoL is a validated questionnaire to retrospectively assess patients’ QoL in the past 4 weeks. It consists of 17 questions from four domains functioning, fatigue/mood, fears/shame, and nutrition and is calculated by using linear transformations of its raw scores to a 0 to 100 point scale, with high values indicating a high degree of quality of life impairment (12, 13).

## Results

### Most patients continue their LTP with lanadelumab and use injection intervals of 30 days or longer

**Figure 1:**
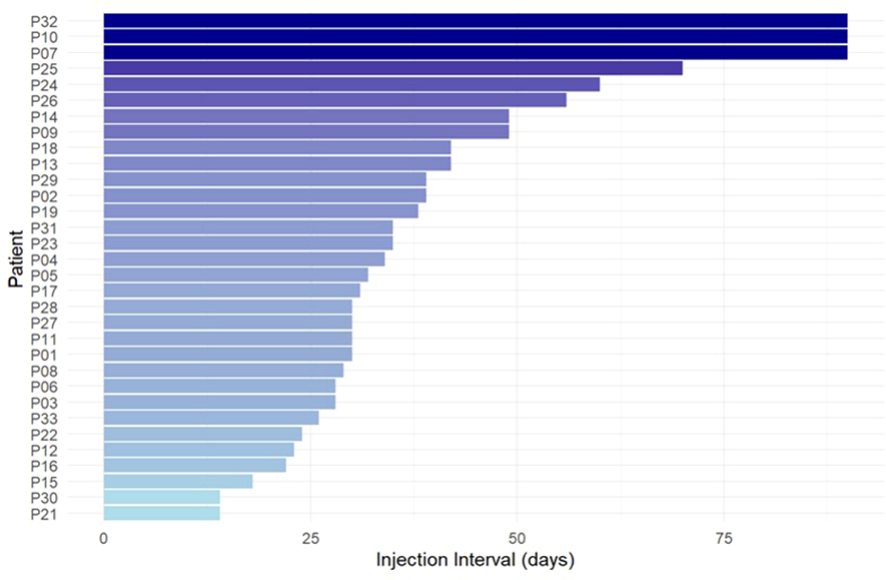
Current injection interval of lanadelumab for each patient.

Across 32 patients, the median injection interval of lanadelumab was 33 days (range 14–90 days, **Figure 1**). Patients with HAE-C1INH reported longer median injection interval (n=28, 35 days, range 14-90 days) than patients with AAE-C1INH (n=4; 23 days, range: 14-31 days).

All patients identified their current dosing interval with the help of the Berlin protocol, i.e. by increasing the interval with complete response and shortening the interval when prodromes or breakthrough attacks occurred (9). The current injection interval, across all patients, has been used for a median period of 27 months (range 4-72, mean 29 ± 17). All patients were satisfied with finding their individual lanadelumab injection interval duration with the help of the Berlin protocol. In contrast, no patient would have preferred a fixed 14-day interval without individual adjustment.

### Lanadelumab leads to long-lasting freedom from attacks in real-life

Since their initiation of lanadelumab, patients, on average, had 2 angioedema attacks (median, range 0-15, mean: 2.54). Patients with HAE-C1INH reported lower median attack rates (2, range 0-8) than patients with AAE-C1INH (6, range 0-15). Across 50 ± 5 months of average lanadelumab treatment (excluding 4 patients who were roll-overs from the open-label-extension HAE LTP study (7)), yearly attack rates were 0.620 in patients with HAE-C1INH and 1.56 in patients with AAE-C1INH. Four and one patient with HAE-C1INH and AAE-C1INH, respectively, have been attack-free since they started lanadelumab treatment.

For their current injection interval, patients reported an average of 0.97 attacks (median 0, range 0-8) during a mean time period of 29 months, i.e. a yearly attack rate of 0.338 ± 0.12. Patients with HAE-C1INH and AAE-C1INH reported an average of 0.71 and 2.75 attacks, respectively, with their current injection intervals (average duration 28.5 months and 30.3 months, respectively). This corresponds to a yearly attack rate of 0.254 and 0.925, respectively.

Since the start of their current injection interval, 23 patients have been completely attack-free including 2 patients with AAE-C1INH. The longest absence of attacks since the initiation of lanadelumab was 24 months (median, range 6-58) and 16 months (median, range 6-45) in HAE-C1INH and AAE-C1INH patients, respectively.

### Lanadelumab continues to improve disease control and quality of life over time

At the time of assessment, all patients had well-controlled disease, i.e. an AECT score of ≥ 10 of 16 points. The median AECT score of all patients was 16 points (range 14-16, mean 15.5 points), and 21 patients scored 16 points, i.e. had complete disease control. The mean AECT score values increased by 0.81 points as compared to those of 3 years ago.

**Figure 2:**
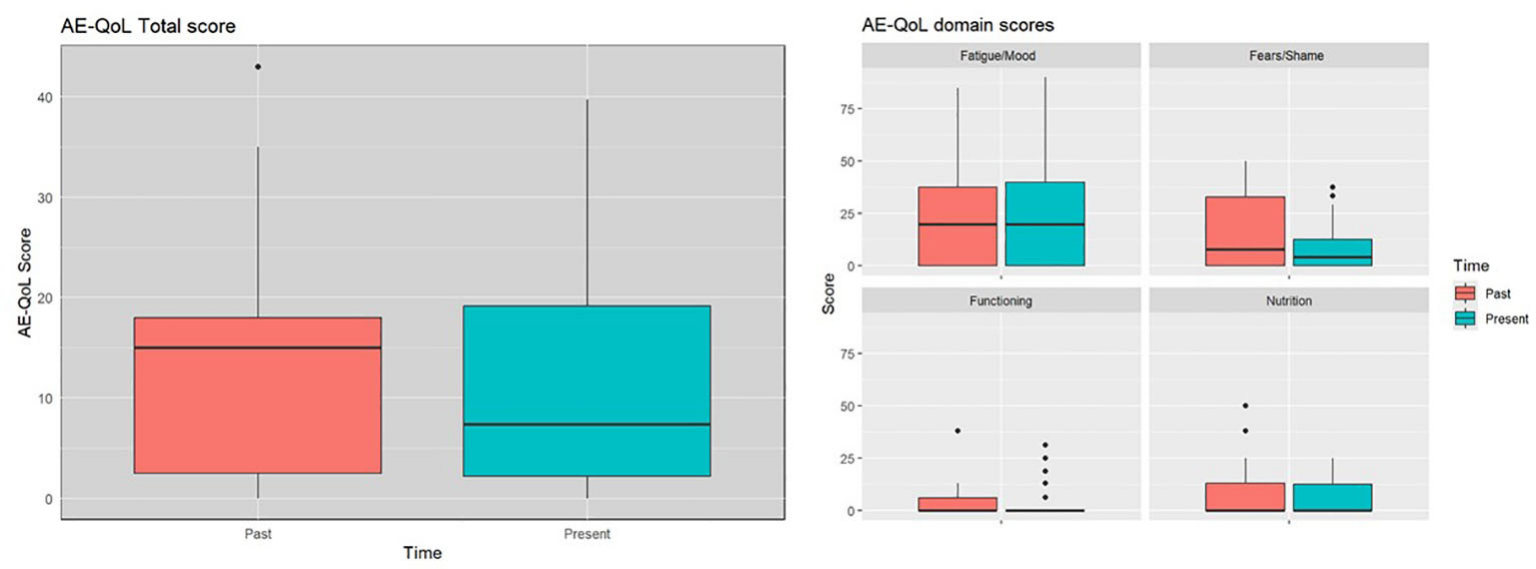
Past and present AE-QoL total scores and domain scores.

**Figure 3:**
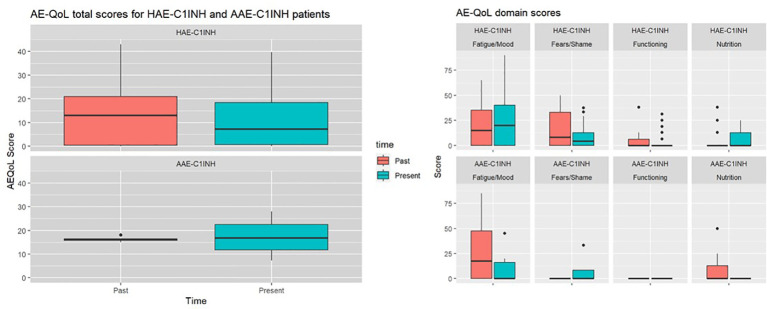
Past and present AE-QoL total values and its domains in patients with HAE-C1INH (n=28) and patients with AAE-C1INH (n=4).

The mean AE-QoL total score was 12 ± 11 points, two points lower than 3 years ago. The largest reduction, was observed in the fears/shame domain. The mean value for the domain functioning, fatigue/mood, fears/shame and nutrition was 3 ± 8, 24 ± 26, 10 ± 12, and 6 ± 10 points, respectively (**Figure 2**). Patients with HAE-C1INH showed lower mean AE-QoL total values than patients with AAE-C1INH (10.8 ± 11 vs. 17.3 ± 9, respectively) and lower mean domain scores for functioning, fatigue/mood, fear/shame, and nutrition (2 ± 6 vs 9 ± 15; 23 ± 27 vs 26 ± 17; 8 ± 12 vs 18 ± 11 and 6 ± 10 vs. 9 ± 12 points, respectively) (**Figure 3**).

### The majority of patients report no or localized mild side effects

Lanadelumab was well tolerated in all patients. Localized burning sensation and itching at the injection site was reported by four patients and one patient, respectively. One patient with AAE-C1INH reported occasional flu-like symptoms for 2-3 days after the injection of lanadelumab.

When patients were asked whether they felt that lanadelumab had become less effective over time, three patients answered yes, with two of these patients belonging to the group that had to shorten the injection interval again in the past. Two patients stated that ODT with C1INH or icatibant was less or slower effective than before LTP with lanadelumab.

### Lanadelumab prevents angioedema attacks due to medical procedures and exposure situations, even without prior use of short-term prophylaxis

In total, 22 patients reported 147 medical procedures and/or exposure situations for increased risk of the occurrence of angioedema attacks, e.g. tooth extractions, dental implants, laryngeal intubations and gastroscopies since the initiation of lanadelumab. Even though sufficient C1INH is available to all patients on request for STP before medical procedures, only two patients used STP with pdC1INH for all procedures they underwent.

In 146 of 147 exposure situations, no angioedema attack occurred on lanadelumab LTP without additional STP (**Table 1**).

**Table 1:** Reported medical procedures under lanadelumab treatment without prior use of short-term prophylaxis treatments.

	Number of patients	Number of events	No angioedema	Angioedema
**Tooth extraction**	4	12	11	1
**Dental implant (procedure on the jawbone)**	2	6	6	0
**Dental cleaning/periodontal disease prevention**	12	45	45	0
**Root canal treatment/crown/bridge insertion**	7	14	14	0
**Drilling and filling of teeth**	13	31	31	0
**Other dental measures**	7	25	25	0
**ENT procedure**	1	1	1	0
**Intubation**	3	5	5	0
**Gastroscopy**	6	8	8	0
**Total**		**147**	**146**	**1**

In one case, swelling of the mouth and neck occurred after the extraction of four teeth without prior STP. In addition, in a patient with 24 tattoos, 21 of these tattoos obtained before the start of lanadelumab each resulted in angioedema. The three other tattoos were done during treatment with lanadelumab and none of them resulted in angioedema. Of the two piercings she has, one was pierced before and one after the initiation of lanadelumab. The first one, but not the second one triggered an angioedema attack.

### Satisfaction with the treatment

**Figure 4:**
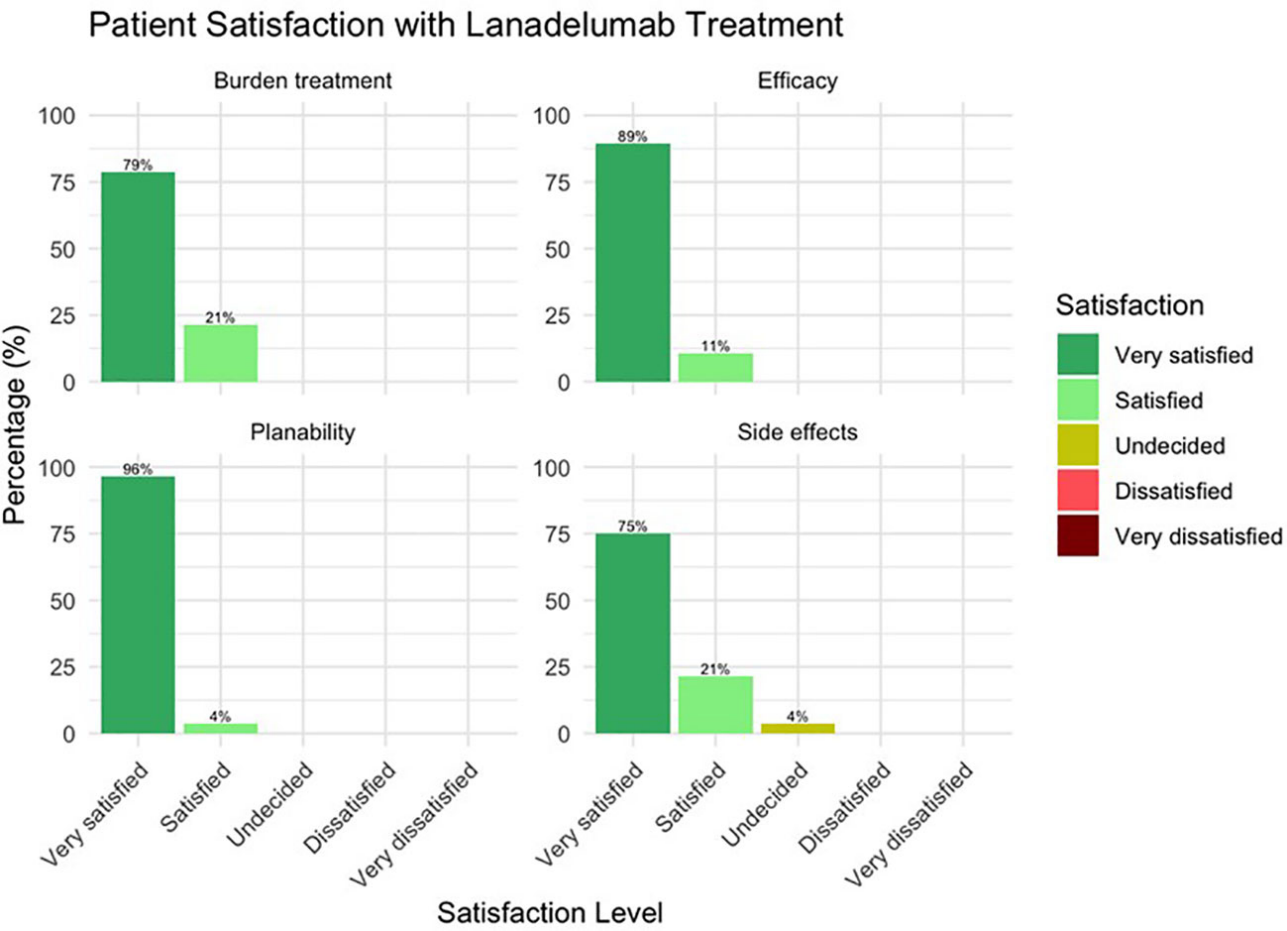
Treatment satisfaction with lanadelumab after an average treatment period of 50 months.

In response to the question “How satisfied are you with your therapy with lanadelumab with regard to a) the efficacy b) the predictability of the therapy c) the extent of the burden of the therapy itself and d) the occurrence or absence of side effects, all patients responded with “very satisfied” or “satisfied”. Only one patient was “undecided” with regard to satisfaction with safety (**Figure 4**).

## Discussion

The results of this real-life study underline the excellent long-term efficacy and safety profile of lanadelumab as LTP in patients with HAE-C1INH and AAE-C1INH over a period of more than 4 years. Almost all patients, 32 of 34, continued the treatment without interruption. The two patients who discontinued the drug did it on their own request. In the current evaluation, the median injection interval of 33 days increased even further as compared to our initial report. Moreover, the yearly mean attack rate of 0.697 over an average time period of 50 months lanadelumab treatment resulted in further improvement in both patients’ disease control and quality of life.

At the time of the evaluation, all but one patient used a fixed injection interval. The extension of the injection interval is generally possible according to the patient information leaflet, but is limited to a maximum interval of four weeks especially in lightweight patients. In the current study, we did not record the body weight of the patients, but there was no obvious indication of a connection between the current injection interval and the body weight of the patients. We rather saw differences in the individual injection intervals between patients with HAE-C1INH and AAE-C1INH, but these observations should be confirmed on larger samples with AAE-C1INH.

The potential of lanadelumab for extending the interval was quickly recognized and applied by many physicians (14–17). The largest data collection to date in this context is the INTEGRATED study, in which 198 patients with HAE from Germany, France, Austria and Greece were analyzed (18). In this retrospective study, 98% of the patients started lanadelumab with a 2-week interval, but after an average of 8 months, 73% were adjusted to a longer interval, and 44% to a 4-week interval. The mean monthly attack frequency in these patients was 0.12 attacks per person and the cumulative AFR (attack free rate) was 54.4%. In our study, we calculated a monthly attack rate of 0.021 for the HAE patients in their current dosing interval. Accordingly, the cumulative AFR for the current interval in our study was 82%, i.e. 23 of 28 HAE patients were symptom-free for an average period of 29 months of constant treatment interval. We suggest that the better performance of our patients is not least due to the use of the Berlin protocol for lanadelumab (9). This strategy allows both physicians and patients to work out the best individual dosing interval in a structured way and then adjust it if necessary, which ultimately leads to an optimal ratio of effectiveness and burden of the therapy compared to the injection of a 14 or 28-day interval. At the same time, this approach optimizes the economic aspect of the treatment.

In terms of disease control, we even observed further improvement in our cohort when compared to our previous report and other studies published: 66% of patients in the current analysis vs. 65% in our initial report showed complete disease control (AECT = 16 points), which exceeds the rate of patients with complete disease control (51%) from the open-label-extension HAE LTP study (6). Additionally, our cohort further improved in the AE-QoL values, albeit only by two points, but the averaged total scores (12 points) were significantly lower than reported by Lumry et al. (21 points) (6). Of note, changes in the fear/shame domain in particular showed a striking result with a clinically significant reduction in the score from 16 to 10 points. From our experience through the routine use of the AE-Qol we have been making this observation of the delayed improvement in the fear/shame domain for some time in patients who were switched to modern LTP treatment options, but we have never been able to demonstrate this as clearly as in this study. Our results show, that patients’ fear of breakthrough attacks is deep-seated and building confidence in a new treatment to finally reach the ultimate treatment goal of “normalization patients` lives” takes a long time.

With regard to adverse events, the current observation cannot contribute much new information: Apart from the well-known local burning sensation on the injection site, no new treatment-related adverse events were reported from our cohort. Of some interest may be the information from one patient who occasionally reported flu-like symptoms two to three days after the injection of lanadelumab. However, such mild temporary side effects are also described from the use of other monoclonal antibodies in rare cases (19). Sporadically, we hear the question from patients and colleagues whether the efficacy of lanadelumab may diminish or whether the effect of ODT diminishes under LTP. Only three patients reported such an impression. It should be taken into account that the administration of ODT has become a rare event for many patients under LTP with lanadelumab and that there may therefore be a recall bias, in the sense that a rare sudden acute attack is perceived as a more threatening and more difficult to control event than when the last attack was still well remembered.

Satisfaction with the therapy appears to be very good overall, with the vast majority of patients being very satisfied with the effectiveness, plannability, burden caused by the treatment and safety. Three of the seven patients who said they were “satisfied” or “undecided” with lanadelumab regarding side effects did not mention any when asked. Possibly, fear of side effects may also lead to a reduction in satisfaction. Taking into consideration the intravenous past, the burden of treatment can currently be categorized as comparatively low, although an oral LTP, i.e. the kallikrein inhibitor berotralstat, has been available for some time, the burden of which is perhaps even lower (20, 21).

The need for STP under complete response to LTP is controversially discussed. On the one hand, there is an interest in minimizing risk and avoiding avoidable complications, both medical, emotional and legal. On the other hand, there is the observation that no natural everyday trigger has been able to cause an attack, raising the question of why an iatrogenic intervention should be able to do this. The almost complete lack of published data on this topic made an end to this debate seem elusive. All patients were offered a prescription for STP, but many of them rejected it. We did not encourage our patients, but over time and with growing experience we stopped enforcing STP on our patients. This resulted in data of 20 patients reporting 147 individual procedures without prior STP, most of which were worthy of using it. Importantly, in 146 of 147 procedures no attacks happened. It is appropriate to shed some light on the one case where an attack followed a procedure. This case was reported by a patient with AAE-C1INH without evidence for underlying lymphoproliferative disease despite regular hemato-oncological presentations. After the initiation of lanadelumab in 2019, two attacks occurred, while the injection interval was extended to up to 44 days. In 2020 there were more breakthrough attacks, some unexpectedly in the middle of the interval, so that the interval was initially reduced to 26 days and later to 23 days. Furthermore, there was one attack and two attacks in 2021 and 2022, respectively. Despite the unchanged general conditions, the number of attacks increased again in 2023, which is why the interval was shortened again to 19 days. 19 days after the last injection of lanadelumab, the patient reported swelling of her lip and neck after four teeth were extracted the day before without prior administration of STP. The use of ODT with icatibant in this case was successful. The course of this patient is unusual in many respects. In no other patient did the injection interval have to be readjusted so frequently and yet no permanent freedom from attacks could be achieved. The patient achieved in the AECT the lowest score in the cohort (12 points) and in the AE-QoL the second highest score (28 points). Thus, it is not surprising that the only failure of LTP was for an iatrogenic trigger in this patient. There is relatively little comparative data to inform the debate about the need for STP in patients with LTP. Bork et al. reported in 2011 from 171 patients who underwent the extraction of one or more teeth a total of 705 times (22). Tooth extractions without previous STP led to subsequent swelling in 21.5% of cases. Pre-procedural administration of 500 units of C1INH reduced the rate to 16%; and with 1000 units, to 7.5% of cases. However, the extent to which these reported patients used LTP at the time of tooth extraction was not reported. In 2017, a registry reported that a failure rate of 4% was observed for STP with pdC1INH up to the day after the procedure, but the exposure situations or procedures have not been specified (23). In our study, the failure rate across all procedures and tooth extractions was 0.7% and 8.3%, respectively. Nevertheless, due to the small sample size, these data must be interpreted with caution.

The introduction of lanadelumab as LTP has significantly changed the therapeutic landscape in C1INH deficiency. The results of this study prove the long-term efficacy and safety of lanadelumab in real-life, which may serve as a benchmark for other emerging treatment options. In view of future developments and the current clinical trials, it is likely that the use of LTP will be further strengthened. Recommendations on the usefulness of STP under effective LTP should be re-assessed.

## Data availability statement

The raw data supporting the conclusions of this article will be made available by the authors, without undue reservation.

## Ethics statement

This retrospective chart review was conducted at the Institute of Allergology IFA of the Charité University Hospital. The data were collected in line with the Landeskrankenhausgesetz (LKG) §25 as part of routine treatment, ensuring anonymity of the patient in the following data review. The studies were conducted in accordance with the local legislation and institutional requirements. The participants provided their written informed consent to participate in this study. Written informed consent was obtained from the individual(s) for the publication of any potentially identifiable images or data included in this article.

## Author contributions

TB: Conceptualization, Writing – original draft, Writing – review & editing, Methodology, Validation. CV: Writing – original draft, Data curation. SA: Data curation, Writing – original draft. KW: Data curation, Writing – original draft. AG: Data curation, Writing – original draft, Visualization. MMau: Conceptualization, Supervision, Writing – original draft, Writing – review & editing. MMag: Conceptualization, Methodology, Supervision, Writing – original draft, Writing – review & editing.
